# Annals of the Medical Society of the County of Albany from 1860

**Published:** 1864-02

**Authors:** 


Annals of the Medical Society of the County of Albany from 1860.—J. Munsell, of Albany, proposes to publish, if a subscription of 100 copies can be obtained, the Minutes of the Albany County Medical Society, from the period of its organization, during about half a century, embracing all which has been preserved that relates to its history and progress. The work will also contain biographical notices of deceased members, and will be a desirable volume to all who feel an interest in matters pertaining to their profession which have transpired in that vicinity, and in which many of them have been identified.

The work will be edited by Dr. S. D. Willard, and will contain about 300 pages in octavo; it will be printed on good paper and bound in cloth, at $2 50.

Those who desire copies upon the above terms, payable on delivery, are requested to forward their names.

